# Pulpotomy for teeth with irreversible pulpitis in immature permanent teeth: a retrospective case series study

**DOI:** 10.1038/s41598-024-56975-6

**Published:** 2024-03-16

**Authors:** Na Zhang, Qian Kang, Yuzhao Cheng

**Affiliations:** Department of Oral Medicine, Shanxi Provincial People’s Hospital, Taiyuan, 030012 China

**Keywords:** Pulpotomy, Dental caries, Tooth root, Pulpitis, Case series, Diseases, Medical research

## Abstract

To evaluate the success of pulpotomy in treating immature permanent teeth with irreversible pulpitis. This case series included patients with irreversible pulpitis admitted to the Department of Oral Medicine at the author’s Hospital between 2015 and 2020. The pulpotomies were carried out by clinicians with > 5 years of working experience. The follow-up findings and radiographic images were reviewed by two attending dentists. This study included 49 teeth from 48 children (25 boys and 23 girls). The follow-up was 23.3 ± 6.8 months (from 12 to 40 months). The success rate of pulpotomy was 85.7% (42/49). Pulpotomy failed in seven teeth (14.3%). The treatment success rate for traumatic crown fracture was lower than for dental caries and dens evaginatus (P < 0.001). There were no significant differences in the success rate of the pulp-capping agent, tooth root developmental phase, and pulpotomy method (all P > 0.05). Pulpotomy might be successfully used to treat immature permanent teeth with irreversible pulpitis in young patients mainly caused by caries and a fractured tubercle of dens evaginatus.

## Introduction

Decay of permanent teeth is a common disease, affecting more than 2.5 billion people worldwide^1^. Irreversible pulpitis is the natural consequence of untreated tooth decay and refers to dental pulp inflammation induced by trauma, caries, or a fractured tubercle of dens evaginatus^2^. Untreated irreversible pulpitis in young permanent teeth can also lead to tooth developmental arrest, a thin root canal wall, and a disproportionate crown-to-root ratio, increasing the risk of secondary root fracture^3^. Nevertheless, no universal standards exist for treating irreversible pulpitis in immature permanent teeth^4,5^.

The American Association of Endodontists recommends total pulpectomy plus root canal filling for irreversible pulpitis^6^. Apexification is another popular strategy to treat irreversible pulpitis in immature teeth^7^. Still, both procedures involve completely removing the radicular pulp, which can cause a thin root canal wall and a disproportionate crown-to-root ratio, and they increase the risk of secondary root fracture. Pulpotomy is an effective alternative for treating traumatic pulp exposure, asymptomatic carious pulp exposure, and reversible pulpitis in permanent teeth^8,9^. Only the infected coronal pulp is removed during pulpotomy, and the non-infected root pulp is preserved. The apical foramen of young permanent teeth is larger, and the pulp tissue at the apex is papillary and migrates with the underlying periapical tissue. The local blood microcirculation system is rich, which also gives the pulp of young permanent teeth a strong defense against inflammation, providing a physiological basis for preserving young permanent teeth with vital pulp^10^. Nevertheless, young permanent tooth pulp tissue has less peritubular dentin and secondary dentin, high dentin permeability, rapid progression of external stimuli, and strong harmful stimuli to the pulp. In addition, the inflammatory manifestations of young permanent teeth are more pronounced than those of mature permanent teeth^11^. Therefore, a different treatment should be considered when treating young permanent teeth.

In recent years, there has been increasing interest in treating irreversible pulpitis using pulpotomy^12–14^. In mature teeth, newly developed biomaterials and increased knowledge of pulp biology have increased the success rates for pulpotomy, even in treating irreversible pulpitis^15^. Nevertheless, there is still little data available on the outcomes of pulpotomy in immature permanent teeth. The American Academy of Pediatric Dentistry (AAPD) guidelines (2020) mentioned only irreducible pulpitis among the indications for pulpectomy in young permanent teeth^4^. Apexification and regenerative endodontics are treatments performed after pulp removal to enhance root growth^4^. The intracanal materials used in apexification to induce or create an apical calcified barrier can be traditional calcium hydroxide^16^. Vitapex is a commercial mixture of calcium hydroxide, iodoform, silicone oil, and other compounds (proprietary composition) administered using a syringe^17^. Other formulations of calcium hydroxide, iodoform, and silicone oil are also available (e.g., Metapex and CHPPs)^16^. Calcium hydroxide for apexification can control inflammation and infection and induce a calcified barrier at the apex of immature teeth with pulp necrosis^16^. The iodoform has bacteriostatic properties through free iodine release, and viscous/oily vehicles prolong the action of calcium hydroxide^17^. Despite the recent development of calcium silicate-based materials (e.g., mineral trioxide aggregate (MTA) and Biodentine) for the treatment of immature permanent teeth with pulp necrosis and apical periodontitis, calcium hydroxide and modified calcium hydroxide paste (e.g., Vitapex) are still widely used due to their commercial availability^16^. Regardless of the disadvantages of extended treatment time and risk of root fracture with calcium hydroxide apexification, the outcomes, including clinical success rate, radiographic success rate, and apical barrier formation rate, are comparable between calcium hydroxide and MTA^16^. Calcium hydroxide apexification performed better than MTA apexification for the elongation of root length^16^.

Therefore, this study aimed to evaluate the success of pulpotomy in treating immature permanent teeth with irreversible pulpitis and present the clinical experience of our center.

## Methods

### Study design and population

This case-series study included patients with irreversible pulpitis admitted to the Department of Oral Medicine at the author’s Hospital between March 2015 and November 2020. The inclusion criteria were (1) age 6–14 years, (2) diagnosis of irreversible pulpitis of immature permanent teeth in Nolla stage 7, 8, or 9^4,18^, (3) underwent pulpotomy, and (4) follow-up was at least 12 months. The exclusion criteria were (1) the clinical manifestation suggested reversible pulpitis or periapical periodontitis or (2) incomplete clinical data.

The diagnostic criteria of irreversible pulpitis included (1) spontaneous pain, night pain, persistent pain, or pain on biting and chewing after cold and heat stimulation, (2) clinical examination with percussion pain or dental pulp temperature test can cause sustained pain, and (3) radiograph examination of apical peritubular dentin gap was normal or widened, or small area low-density shadow was seen^19^. A tooth had to meet all inclusion criteria and at least one criterion of irreversible pulpitis to be included in the study.

This study was approved by the Ethics Committee of the author’s Hospital. Given the retrospective nature of the study, the requirement for informed consent was waived.

### Surgical procedures

The pulpotomies were carried out by clinicians with > 5 years of working experience. Local anesthesia was applied around the affected tooth by injecting 1:100,000 epinephrine hydrochloride and mepivacaine (20.00 mg/ml mepivacaine hydrochloride + 0.01 mg/ml epinephrine). Under protection from a rubber dam isolation, the fast emery drill was used to remove the surface enamel, and the slow-speed tungsten steel ball drill or spoon excavation was used to remove the dentin caries and expose the pulp. First, the status of the pulp and bleeding was assessed. Next, a sterile high-speed diamond ball drill was used for partial or total coronal pulp removal; partial or total pulpotomy was performed depending on the bleeding of the pulp after pulpotomy (if the bleeding could not be controlled after partial pulpotomy, deeper areas of the pulp would be removed). Then, 2.5% sodium hypochlorite was used for rinsing, and a sterile saline cotton ball was placed in the pulp section for compression for 3–5 min. After removing the cotton ball, the pulp section without active bleeding was considered adequate hemostasis. Then, MTA (Proroot MTA, Densply, USA) or modified calcium hydroxide paste (Vitapex, Morita, Japan) 2–3 mm thick was applied. Light-cure glass ionomer (Fuji II, GC, Japan) was applied as a base, and composite resin (Tetric N-Ceram, Ivoclar Vivadent, Liechtenstein) was used to repair the enamel. There were no criteria for the selection of MTA or Vitapex. Vitapex was used in most of the early cases, while the use of MTA gradually increased and was more common in the latter cases.

### Data collection

The clinical characteristics of the patients were extracted from the medical records. The clinical characteristics included age, sex, type of traumatic injury, pulp-capping agent, tooth root development phase, method of pulpotomy of coronal pulp, dentin bridge, time of follow-up, conversion to other treatments, and success rates. Two attending dentists reviewed the follow-up findings and radiographic images. The radiographic images were reviewed by each investigator once, re-reviewed again 2 weeks later, and the results were reported. Any inconsistency found during the review was resolved through discussion.

A case was considered clinically successful if there was no history of spontaneous pain or discomfort (except during the first few days after treatment), the tooth was functional, and there was no pain or discomfort when chewing or eating, there was a positive response to the cold test, no tenderness to percussion or palpation, normal grade I mobility, and the soft tissues around the tooth were normal with no swelling or sinus tract. The case was considered radiographically successful without internal or root resorption. Persistent severe spontaneous pain, tenderness to percussion, development of a sinus tract, swelling, or a negative response to cold testing was considered a clinical failure; intraradicular or extraradicular pathosis on the recall radiograph was considered a radiographic failure, and root canal treatment was initiated in such cases^20^.

### Statistical analysis

The Shapiro–Wilk test was performed to test the continuous data for normal distribution. Normally distributed continuous data were described as mean ± standard deviation. Continuous data conforming to a skewed distribution were described as median (range). Categorical data were described as n (%). Analysis of variance (ANOVA) was used to analyze normally distributed quantitative data. The Kruskal–Wallis test was used to test the non-normally distributed continuous data. The chi-square test and the Fisher exact test were used for categorical data. All statistical analyses were performed using SPSS 22.0 (IBM, Armonk, NY, USA). Two-sided P-values < 0.05 were considered statistically significant.

### Ethics approval and consent to participate

All procedures were performed in accordance with the ethical standards laid down in the 1964 Declaration of Helsinki and its later amendments. This study was approved by the Ethics Committee of Shanxi Provincial People’s Hospital [(2021) No. 249 of Shanxi medical ethical approval]. The requirement for individual Informed consent was waived by the Ethics Committee of Shanxi Provincial People’s Hospital because of the retrospective nature of the study. The study was carried out in accordance with the applicable guidelines and regulations.

## Results

This study included 48 patients with irreversible pulpitis, 25 boys and 23 girls, aged 9.5 years (range, 8.28–11 years). Among the 48 patients, 49 teeth were treated, including 28 teeth with caries, 15 with dens evaginatus, and six with traumatic crown fracture. Thirty-nine teeth underwent total pulpotomy of the coronal pulp, and 10 underwent partial pulpotomy of the coronal pulp. Vitapex or MTA was used as the capping agent in 17 and 32 teeth, respectively. The follow-up was 23.3 ± 6.8 months (range, 12–40 months) (Table 1). Supplementary Table S1 presents the characteristics of each patient.

**Table 1:** Baseline characteristics of the patients.

Variables	Patients (n = 48)
Sex	
Male	25 (52.1%)
Female	23 (47.9%)
Age (years), (mean, range)	9.5 (8.3,11.1)
Type of traumatic injury	
Dens evaginatus	15 (30.6%)
Dental caries	28 (57.1%)
Traumatic crown fracture	6 (12.2%)
Pulp-capping agent	
Vitapex	17 (34.7%)
MTA	32 (65.3%)
Stage of tooth root development	
7/8	20 (40.8%)
9	29 (59.2%)
Method of pulpotomy	
Partial	10 (20.4%)
Total	39 (79.6%)
Time of follow-up (months), (mean ± SD)	23.3 ± 6.8
Dentin bridge	
No	19 (38.8%)
Yes	30 (61.2%)
Outcome	
Success	42 (85.7%)
Failure	7 (14.3%)

The number of patients was 48, and the number of treated teeth was 49. The values associated with age and observation time were calculated according to the number of patients; the values associated with the remaining characteristics were calculated according to the number of teeth.

Among the 28 patients with dental caries, eight underwent partial pulpotomy, while the others underwent total pulpotomy. All six patients with traumatic crowns had enamel-dentin fractures.

According to the number of teeth, the treatment success rate was 85.7% (42/49). The treatment failed in seven teeth (14.3%). The treatment success rate for traumatic crown fracture (33.3%) was significantly lower than for dental caries (89.3%) or dens evaginatus (100%) (P < 0.001) (Table 2). The Supplementary Materials present a typical case. Regarding the traumatic cases, Case 6 (21) failed to seek medical attention in a timely manner after a crown fracture; 3 months after injury, dental discomfort occurred, and dental pulp treatment was sought. In case 10 (21), after the initial diagnosis of crown fracture, the enamel fracture surface was covered. After 2 months of observation, discomfort occurred, and pulp treatment was performed. In case 23 (11), after the initial diagnosis of a fractured crown, the crown was fractured and reattached; after 6 months of treatment, tooth discomfort occurred, and pulp treatment was performed. In case 28 (12), after the initial diagnosis of crown fracture, the glass ion fracture surface was covered; after 3 months of observation, discomfort occurred, and pulp treatment was performed. Case 34 (11) failed to seek medical attention in a timely manner after tooth crown fracture and seeking dental pulp treatment after experiencing discomfort one month after injury. Finally, in case 43 (11), after a crown fracture, the patient did not seek medical attention in a timely manner; 1 month after injury, the patient experienced dental discomfort and sought dental pulp treatment.

**Table 2:** Comparison of the failure/success rates among clinical characteristics.

Variable	Failure (n = 7)	Success (n = 42)	P
Disease cause			< 0.001
Dens evaginatus	0 (0.0%)	15 (100%)	
Dental caries	3 (10.7%)	25 (89.3%)	
Traumatic crown fracture	4 (66.7%)	2 (33.3%)	
Pulp-capping agent			0.178
MTA	3 (9.4%)	29 (90.6%)	
Vitapex	4 (23.5%)	13 (76.5%)	
Tooth root developmental phase			0.123
7/8	1 (5.0%)	19 (95.0%)	
9	6 (20.7%)	23 (79.3%)	
Method of pulpotomy			0.148
Partial	0 (0.00%)	10 (100%)	
Total	7 (18.0%)	32 (82.0%)	

## Discussion

Lingering pain is generally believed to be a manifestation of irreversible pulpitis, which does not necessarily imply that the entire pulp is in an irreversible inflammatory state. When lingering pain occurs in the affected teeth, irreversible inflammation may only be present in the coronal pulp but not in the root pulp^21^. Therefore, removing all the pulp for apexification or root canal therapy is not recommended for immature teeth. In recent years, studies have started to focus on the conservative treatment of irreversible pulpitis to preserve the physiological functions of the pulpo-dentinal complex. For the diagnosis of young permanent dental pulpitis, similar to mature permanent teeth, evaluating the child’s medical history, performing a clinical examination and X-ray interpretation of the affected tooth, and carrying out an intraoperative reevaluation of the pulp status before surgery are very important. Although it has been argued that methods based on clinical signs or symptoms are relatively crude and do not reflect the true histopathological state of the pulp^22^, a recent study confirmed a strong correlation between clinical signs and symptoms of reversible/irreversible pulpitis and the histological state of the pulp^21^. New diagnostic classifications have also been proposed, combining diagnosis with treatment, using diagnostic terms such as mild, moderate, and severe pulpitis^23^.

A previous study reported using calcium hydroxide as a capping agent was considered the standard gold treatment for pulp diseases^24^. In addition, the success rate of calcium hydroxide capping ranges from 56 to 74%^25^, similar to the present study that reported a success rate of 76.5% for pulpotomy. Compared with calcium hydroxide, MTA has better biocompatibility and sealing ability, which might induce the proliferation of dental pulp cells and the formation of a thick, complete dentin bridge with fewer defects. Therefore, using MTA as the pulp-capping agent might increase the treatment success rate^11,26,27^. The Vitapex paste is a pre-mixed paste of calcium hydroxide and iodoform that is widely used as root canal filling material for deciduous molars because its reabsorption rate is similar to that of deciduous roots, and they have strong anti-corrosion properties. The iodoform component of Vitapex possesses antimicrobial effects and may provide a better microenvironment for root development^16^. On the other hand, owing to the improved operating performance of the modified paste, underfillings were reduced, thus occupying the canal space for continued root development. A study showed that in teeth capped with an iodoform-based paste, deposition of mineralized tissue occurred far from the pulp exposure area^28^. The iodoform-based paste also showed excellent biocompatibility with pulp fibroblasts, produced a mild inflammatory reaction, and was well tolerated by periapical tissues^28^.

Nevertheless, the literature is scarce regarding the use of Vitapex paste during pulpotomy in young permanent teeth. A previous study showed that the continuity and morphology of dentin bridges induced by Vitapex in pulp were lower than those induced by MTA^29,30^. Therefore, it can be inferred that the dentin bridges formed by Vitapex are also poorly sealed. This study used Vitapex paste or MTA pulpotomy to treat young permanent teeth with irreversible pulpitis. The overall success rate was 85.7%. The success rate of Vitapex paste as a pulp capping agent was 76.5%, and that of MTA was 90.6%, but the difference between the two was not statistically significant. Therefore, considering that MTA is relatively expensive, Vitapex paste is still an option for this treatment.

Sodium hypochlorite is a potent antiseptic that effectively removes carious dentin debris and bacterial contamination and promotes hemostasis as a pulp-rinsing agent^31^. In addition, sodium hypochlorite is non-toxic and does not interfere with pulp healing. Sodium hypochlorite has been widely used in pulpotomy, especially for teeth with pulp inflammation^31^. Nevertheless, there is still no consensus on the time needed to achieve hemostasis after rinsing. Ricucci et al*.*^32^ found that bleeding ceases 2–3 min later, while Duncan et al*.*^11^ found that bleeding stops within 5 min. Moreover, Bogen et al*.*^5^ found that bleeding stops within 10 min. Another study stated that a history of toothache and pulp sensitivity test results were not significantly associated with the degree of pulp inflammation observed in clinical practice^33^. Therefore, it was suggested that the pulp should be directly examined under a dental operating microscope to ensure that the inflammatory tissues are completely removed and to prevent possible root pulp degeneration^34^. This study used 2.5% sodium hypochlorite to rinse the pulp of all teeth. However, as a retrospective study, the hemostasis time was not precise.

The dentin bridge formation on the treated pulp section indicates viability and pulp healing capacity and reflects the formation of a protective barrier on the pulp. It has been suggested that dentin bridge formation is an important indicator of treatment success^35^. However, Taha et al*.*^36^ assessed the effects of pulpotomy in immature permanent teeth with carious pulp exposure and found dentin bridge formation in 25% of teeth at 1-year follow-up. Mass et al*.*^37^ followed up on the status of the immature permanent teeth treated with partial pulpotomy for carious pulp exposure for 10 years, finding a dentin bridge formation in only 58% of the teeth. The present retrospective analysis of 42 successfully treated teeth also showed dentin bridge formation in 71.4% of teeth (30/42), and treatment success was also achieved in several teeth without dentin bridge formation.

In this study, 16 children (30.6%) had irreversible pulpitis induced by a dens evaginatus; the success rate was significantly lower for traumatic crown fracture, probably due to additional damage to the periodontal supporting tissues. Because all external injuries were non-“fresh”, some patients may experience symptoms during the observation period after treatment, while others may not receive immediate treatment before seeking medical attention. Therefore, the situation in these cases is different from previous literature reports where immediate pulpotomy after trauma can achieve a higher success rate^38^. The lower success rate of treatment can also be related to the failure to detect possible composite tooth injuries in a timely manner during examination^39^. The possible damage to periodontal tissue and the disruption of pulp blood supply in the apical area could be the main reasons for the failure of pulpotomy^40^. Such damage is usually associated with poor blood supply to the tip of the root^40^. It has been reported that pulpotomy generally has a lower success rate for carious teeth than for dens evaginatus or traumatic crowns^41^. On the other hand, the present study showed a relatively high success rate in carious teeth, which is supported by various studies^42–44^. Still, the sample size of patients with trauma was too small to draw conclusions about the possible causes for the low success.

The procedure’s success rate in Nolla stage 9 teeth was similar to that in Nolla stages 7 and 8. Hence, it appears that the stage of root development did not affect the treatment outcome, as supported by previous studies^13,14,45,46^. In some cases, treatment failure might be due to a wrong evaluation of the dental pulp status. Indeed, the actual inflammation of young permanent teeth can be more serious than the symptoms might suggest. Therefore, even in the presence of a relatively complete pulp, irreversible inflammatory reactions can occur.

There were several limitations to this study. First, the data were retrospectively collected from a single center, which might limit the statistical power of the results. Second, the sample size of this study was small, and multivariable regression was not performed to correct for confounding factors. Third, as a retrospective study, the cases in this study may have a deviation in the judgment of the dental pulp state. Therefore, further validation of the results is required in large-scale prospective studies with longer observation times. Furthermore, various aspects of pulpotomy for irreversible pulpitis in immature permanent teeth should be explored in future studies. For example, it could be interesting to conduct further studies to evaluate patients’ perspectives according to the type of anesthesia performed for pulpotomy, particularly in the setting of novel computerized anesthesia devices being developed to improve patients’ experience^47^. Different methods for pulpotomy should also be explored on the patient’s outcomes.

Pulpotomy might be feasible to treat irreversible pulpitis in immature permanent teeth. It might also be suitable for permanent teeth in the early stages of root and irreversible pulpitis caused by caries and dens evaginatus. The irreversible pulpitis in immature permanent teeth does not represent the infection of the entire pulp; the exclusion of infected pulp by pulpotomy might preserve the uninfected pulp.

### Supplementary Information

Supplementary Figure S1.

Supplementary Information.

## Data Availability

All data generated or analysed during this study are included in this published article.
