# Supplementary figures and images for: Pulpotomy for teeth with irreversible pulpitis in immature permanent teeth: a retrospective case series study
Source: Sci Rep. 2024 Mar 16;14:6395. doi: 10.1038/s41598-024-56975-6 (PMC10944512; doi:10.1038/s41598-024-56975-6)

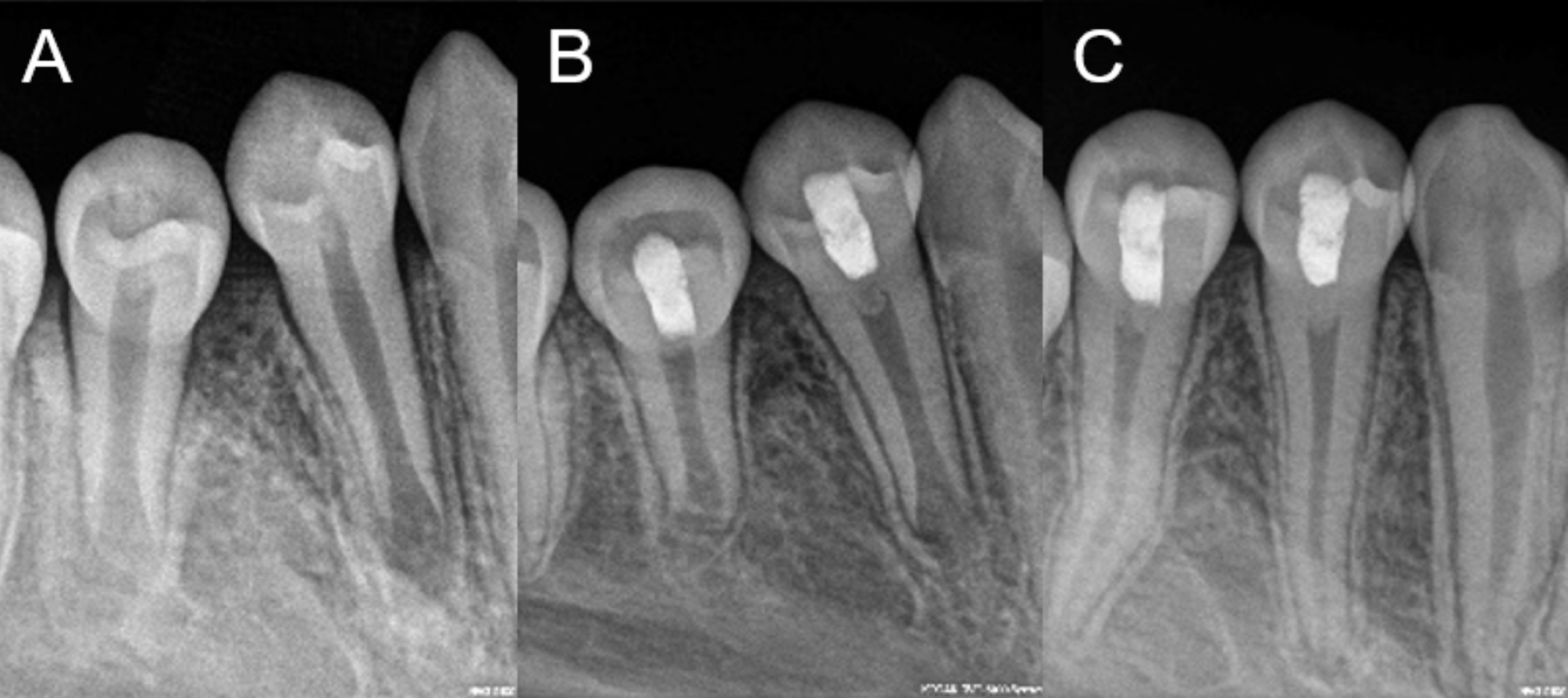

Supplement: Supplementary file 1 — Supplementary Figure S1. [file 41598_2024_56975_MOESM1_ESM.jpg]
