# Supplementary material for: Pulpotomy for teeth with irreversible pulpitis in immature permanent teeth: a retrospective case series study
Source: Sci Rep. 2024 Mar 16;14:6395. doi: 10.1038/s41598-024-56975-6 (PMC10944512; doi:10.1038/s41598-024-56975-6)
Supplement: Supplementary file 2 — Supplementary Information. [file 41598_2024_56975_MOESM2_ESM.docx]

**Supplementary Materials**

**Typical cases**

This patient was 8 years and 11 months old boy who came to the hospital for treatment on December 15, 2015. The patient reported discomfort in two lower right posterior teeth after cold or heat stimulation, which lasted for a few weeks. He experienced pain that affected his sleep during nighttime. The child was healthy, with no family genetic history.

The target sample section was seen on the occlusal surface of teeth 44 and 45. The pain was felt in the center of the affected surface upon examination when stimulated by a cold probe, and the pain continued for several seconds after removing the probe. The teeth were not sensitive to percussion (-), and no obvious abnormalities were seen in the gums.

Radiographs showed that the roots of 44 and 45 teeth were not fully developed (Nolla phase 8), and there were no significant abnormalities in periapical membranous space imaging **(Fig.S1A)**.

The child was diagnosed with acute pulpitis (a fractured tubercle dens evaginatus) of teeth 44 and 45. A rubber dam isolation was placed on teeth 44 and 45 under local anesthesia (mepivacaine was given). When the pulp was opened, bleeding was observed from the coronal pulp. The crown pulp was removed, and 2.5% sodium hypochlorite solution was used for repeated washing until the bleeding stopped. Vitapex paste was used to cover the pulp section, and Fuji II light-cured glass ionomer was placed at the bottom. Selective acid etching of tooth enamel was performed before applying a coated adhesive, and the tooth was then filled with layered TN porcelain resin. Finally, the filling materials were trimmed and polished.

At the 3-month follow-up visit, the patient reported no symptoms of discomfort on the affected side. The filling materials of teeth 44 and 45 were intact. The teeth were not sensitive to percussion (-), and no obvious abnormalities were seen in the gums.

Radiographs showed dentinal bridge development under the pulp cap of teeth 44 and 45, and no significant abnormalities were seen around the apex of the root **(Fig.S1B).**

After 2 years, no discomfort on the affected side was found. The surface of the filling materials of both treated teeth had a slight defect. The teeth were not sensitive to percussion (-), and no obvious abnormalities were seen in the gums. Radiographs showed that the roots of teeth 44 and 45 had fully developed. The apical foramen of the teeth was closed, and the root canal wall was narrowed. Also, no obvious abnormalities were seen around the apex of the root of the teeth. The filled resin in both teeth was removed and replaced **(Fig.S1C)**. The teeth were considered cured.

**Figure Legends**

**Fig.S1 Radiograph examination. A:** Preoperative radiographs: the root development stage was Nolla phase 8, and the periapical membrane cavity was clear. **B:** Calcified bridge formation could be seen 3 months after surgery. **C:** 25 months after surgery, the basic development of tooth roots was completed, the apical foramen was closed, and the wall of the root canal was narrowed.

**Supplementary Table S1.** Characteristics of each included patient

| **No** | **Sex** | **Age (years)** | **Teeth** | **Type of traumatic injury** | **Pulp-capping agent** | **Stage of tooth root development (Nolla stage)** | **Method of pulpotomy** | **Review time (months)** | **Dentin bridge** | **Outcome** |
| --- | --- | --- | --- | --- | --- | --- | --- | --- | --- | --- |
| 1 | Male | 8.2 | 35 | Dental caries | Vitapex | 7 | Total | 26 | Yes | Success |
| 2 | Male | 11.4 | 46 | Dental caries | Vitapex | 9 | Total | 30 | Yes | Success |
| 3 | Female | 9.1 | 25 | Dens evaginatus | Mta | 8 | Total | 23 | No | Success |
| 4 | Male | 8.5 | 45 | Dens evaginatus | Mta | 7 | Partial | 37 | Yes | Success |
| 5 | Female | 12.4 | 47 | Dental caries | Vitapex | 8 | Partial | 19 | Yes | Success |
| 6 | Female | 9.2 | 21 | Dental trauma (enamel-dentin fracture) | Vitapex | 9 | Total | 16 | No | Success |
| 7 | Male | 8.9 | 45 | Dens evaginatus | Vitapex | 8 | Total | 25 | Yes | Success |
|  |  |  | 44 | Dens evaginatus | Vitapex | 8 | Total | 25 | Yes | Success |
| 8 | Male | 9.8 | 46 | Dental caries | Mta | 9 | Total | 20 | No | Success |
| 9 | Female | 7.8 | 36 | Dental caries | Mta | 8 | Total | 40 | Yes | Success |
| 10 | Male | 9.3 | 21 | Dental trauma (enamel-dentin fracture) | Vitapex | 9 | Total | 13 | No | Failure |
| 11 | Female | 10.4 | 45 | Dens evaginatus | Mta | 8 | Total | 14 | Yes | Success |
| 12 | Male | 10.1 | 26 | Dental caries | Mta | 9 | Total | 18 | No | Failure |
| 13 | Female | 12.5 | 45 | Dens evaginatus | Vitapex | 9 | Total | 13 | Yes | Success |
| 14 | Female | 9.6 | 16 | Dental caries | Mta | 9 | Partial | 19 | Yes | Success |
| 15 | Female | 10.9 | 25 | Dens evaginatus | Mta | 9 | Total | 22 | Yes | Success |
| 16 | Female | 8.6 | 36 | Dental caries | Mta | 9 | Total | 12 | No | Success |
| 17 | Female | 14.7 | 17 | Dental caries | Mta | 9 | Partial | 19 | No | Success |
| 18 | Female | 7 | 46 | Dental caries | Vitapex | 8 | Partial | 26 | Yes | Success |
| 19 | Female | 13.2 | 37 | Dental caries | Mta | 9 | Total | 20 | Yes | Success |
| 20 | Male | 14.2 | 27 | Dental caries | Vitapex | 9 | Total | 23 | Yes | Success |
| 21 | Female | 7.3 | 46 | Dental caries | Mta | 8 | Total | 27 | No | Success |
| 22 | Male | 10.2 | 35 | Dens evaginatus | Mta | 9 | Total | 32 | Yes | Success |
| 23 | Male | 8.1 | 11 | Dental trauma (enamel-dentin fracture) | Vitapex | 8 | Total | 25 | No | Success |
| 24 | Female | 13.9 | 37 | Dental caries | Vitapex | 9 | Total | 19 | Yes | Success |
| 25 | Male | 8.2 | 46 | Dental caries | Mta | 9 | Total | 33 | Yes | Success |
| 26 | Male | 8.9 | 36 | Dental caries | Mta | 9 | Total | 25 | No | Success |
| 27 | Female | 11.1 | 35 | Dens evaginatus | Mta | 9 | Total | 23 | Yes | Success |
| 28 | Female | 9 | 12 | Dental trauma (enamel-dentin fracture) | Mta | 8 | Total | 14 | No | Failure |
| 29 | Male | 9.3 | 15 | Dens evaginatus | Mta | 8 | Total | 20 | Yes | Success |
| 30 | Male | 8.1 | 36 | Dental caries | Mta | 8 | Partial | 29 | No | Success |
| 31 | Female | 9.1 | 46 | Dental caries | Vitapex | 9 | Total | 19 | Yes | Success |
| 32 | Female | 11.8 | 37 | Dental caries | Mta | 9 | Total | 21 | Yes | Success |
| 33 | Male | 9.3 | 35 | Dens evaginatus | Mta | 9 | Total | 18 | No | Success |
| 34 | Male | 10.4 | 11 | Dental trauma (enamel-dentin fracture) | Vitapex | 9 | Total | 15 | No | Failure |
| 35 | Male | 9.6 | 46 | Dental caries | Mta | 9 | Total | 30 | Yes | Success |
| 36 | Female | 6.9 | 36 | Dental caries | Mta | 8 | Total | 28 | Yes | Success |
| 37 | Female | 10.1 | 15 | Dens evaginatus | Vitapex | 9 | Partial | 27 | Yes | Success |
| 38 | Male | 12 | 37 | Dental caries | Mta | 7 | Partial | 22 | Yes | Success |
| 39 | Female | 7.8 | 16 | Dental caries | Mta | 8 | Total | 33 | No | Success |
| 40 | Male | 8 | 36 | Dental caries | Mta | 7 | Partial | 35 | Yes | Success |
| 41 | Male | 14 | 17 | Dental caries | Vitapex | 9 | Total | 20 | No | Failure |
| 42 | Female | 11.1 | 25 | Dens evaginatus | Mta | 9 | Total | 19 | Yes | Success |
| 43 | Male | 9.8 | 11 | Dental trauma (enamel-dentin fracture) | Vitapex | 9 | Total | 17 | No | Failure |
| 44 | Male | 6.6 | 46 | Dental caries | Mta | 8 | Partial | 29 | No | Success |
| 45 | Male | 9.4 | 35 | Dens evaginatus | Mta | 8 | Total | 34 | Yes | Success |
| 46 | Male | 13.4 | 37 | Dental caries | Mta | 9 | Total | 15 | No | Failure |
| 47 | Male | 8 | 36 | Dental caries | Mta | 9 | Total | 28 | Yes | Success |
| 48 | Female | 10.5 | 25 | Dens evaginatus | Mta | 9 | Total | 24 | Yes | Success |
